# Thoracic tomographic manifestations in symptomatic respiratory patients with COVID-19

**DOI:** 10.1590/0100-3984.2020.0030

**Published:** 2020

**Authors:** Lucas de Pádua Gomes de Farias, Daniel Giunchetti Strabelli, Eduardo Kaiser Ururahy Nunes Fonseca, Bruna Melo Coelho Loureiro, Cesar Higa Nomura, Márcio Valente Yamada Sawamura

**Affiliations:** 1 Instituto de Radiologia do Hospital das Clínicas da Faculdade de Medicina da Universidade de São Paulo (InRad/HC-FMUSP), São Paulo, SP, Brazil.

**Keywords:** Coronavirus, COVID-19, Multislice computed tomography, Coronavírus, COVID-19, Tomografia computadorizada de multidetectores

## Abstract

China was the epicenter for the novel coronavirus disease (COVID-19), which quickly spread to other Asian countries and later to Western countries; subsequently, COVID-19 was categorized as a pandemic by the World Health Organization. Diagnosis primarily depends on viral detection in respiratory samples; however, available kits are limited, lack high sensitivity, and have a long turnaround time for providing results. In this scenario, computed tomography has emerged as an efficient and available high-sensitivity method, allowing radiologists to readily recognize findings related to COVID-19. The objective of this article is to demonstrate the main tomographic findings in symptomatic respiratory patients with COVID-19 to assist medical professionals during this critical moment.

## Abstract (pt)

A doença pelo novo coronavírus (COVID-19) teve epicentro na China e rapidamente se espalhou pelos demais países asiáticos e, posteriormente, para os países ocidentais, sendo definida como pandemia pela Organização Mundial da Saúde. O diagnóstico da COVID-19 é primariamente dependente da pesquisa do vírus nas vias aéreas superiores, mas os kits para sua confirmação ainda são limitados, não apresentam sensibilidade elevada e os resultados são demorados. Nesse cenário, a tomografia computadorizada surge como método eficiente e disponível e com alta sensibilidade, cabendo a nós radiologistas reconhecer prontamente os achados relacionados a essa doença. O objetivo deste artigo é demonstrar os principais achados tomográficos de tórax em pacientes sintomáticos respiratórios infectados pela COVID-19, de modo a auxiliar os colegas nesse momento crítico.

## INTRODUCTION

Initially, the respiratory infection caused by the novel coronavirus strain SARS-CoV-2, which was first detected in the city of Wuhan (Hubei province, China) in December 2019, was reported as a “pneumonia of unknown etiology”. Since then, it has been named coronavirus disease 2019 (COVID-19) and has rapidly spread worldwide. On March 11, 2020, the World Health Organization declared COVID-19 as a pandemic^(1,2)^.

Reportedly, the tomographic findings of pneumonia caused by COVID-19 are nonspecific and similar to those induced by other viral infections, such as influenza, COVID- 19 triggers pneumonia, drug-induced pneumonitis, and connective tissue diseases^(3)^. Computed tomography (CT), although recognized as an extremely sensitive method, has not been indicated as the first diagnostic approach by several radiology societies, restricting its utility for specific cases such as hospitalized and symptomatic patients or in specific clinical situations^(3-6)^. The reference diagnosis is based on reverse transcription polymerase chain reaction (RT-PCR) and a dissociation between laboratory and tomographic findings may be present even in initially symptomatic patients^(3,7,8)^.

**Figure 1:**
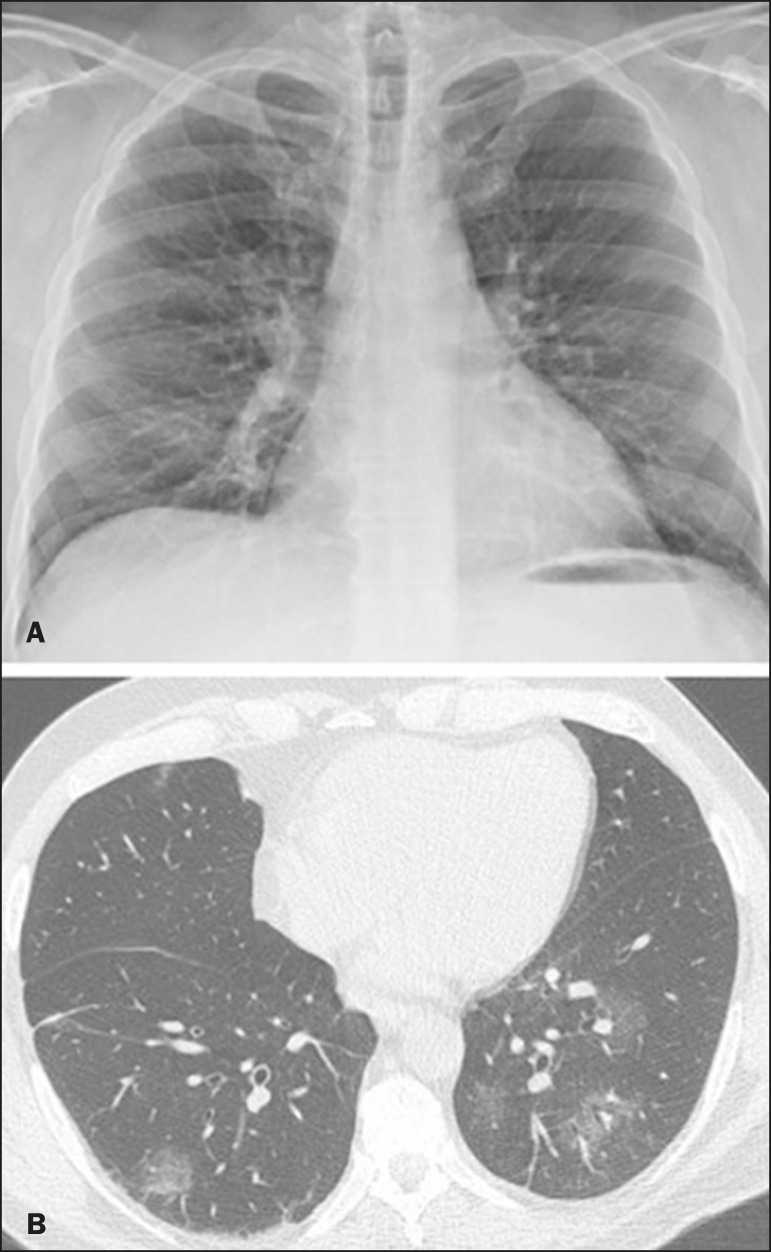
Male patient, 35 years old. Posteroanterior chest radiography (**A**) showing no pulmonary changes. MDCT (**B**) on the same day shows opacities with ground-glass attenuation in the lower lobes. Positive RT-PCR for SARS-CoV-2.

Chest radiography has limited efficiency due to its low sensitivity and specificity, demonstrating normal or ambiguous findings in most initial cases (Figure 1); as such, clinicians and radiologists need to be aware of these limitations^(4,9)^. However, it can be used at bedside and in field hospitals. In contrast, ultrasonography can be used to monitor patients, especially those in intensive care units, as it can identify peripheral changes in the lung, the region most affected by the virus^(10)^.

The objective of this study was to present the most significant imaging results obtained from symptomatic respiratory patients with COVID-19 using a multidetector CT (MDCT). These findings were described according to the Brazilian consensus on the terminology of key descriptors and standards^(11)^.

## PULMONARY MANIFESTATIONS

Parenchymal changes induced by the novel coronavirus are possibly related to its affinity for the angiotensin-converting enzyme 2^(12)^, which is highly expressed in the lungs and heart, allowing the virus to invade alveolar epithelial cells and resulting in cell damage and local inflammatory responses that lead to respiratory symptoms^(13)^. Postmortem histopathological examinations have demonstrated diffuse alveolar damage with fibromyxoid exudates, characterized by pulmonary edema with hyaline membrane-formation and pneumocyte desquamation, which indicate an early phase of acute respiratory distress, suggesting that this is the pathological process underlying the tomographic findings of alveolar and interstitial damage and parenchymal changes^(14)^.

Initially, CT scans may fail to detect changes or indicate only small areas of ground-glass opacity, which are sometimes isolated. Over time, the number and extent of pulmonary findings increase, mainly with consolidations and crazy-paving areas. These findings usually peak around the 10th day and slowly regress. In the late stages, linear opacities are commonly observed, although ground-glass opacities and consolidations may persist^(15)^.

### Ground-glass pattern

**Figure 2:**
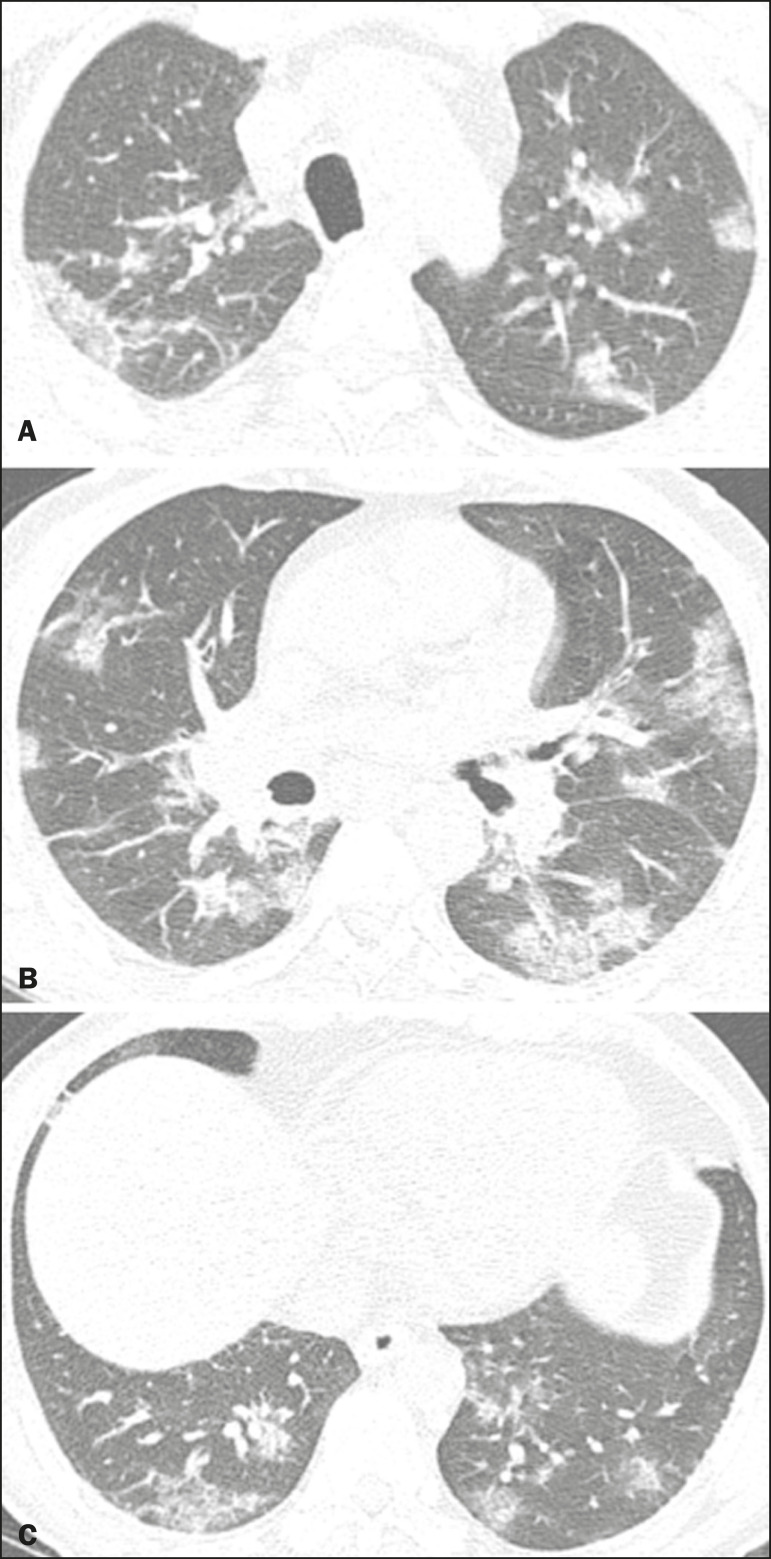
Male patient, 48 years old. MDCT shows ground-glass opacities and diffuse consolidation foci in the upper (**A**), middle (**B**), and lower (**C**) lung fields with predominantly peripheral distribution in the posterior regions. Positive RTPCR for SARS-CoV-2.

A ground-glass pattern is defined as an increased density of the pulmonary parenchyma that does not obscure the bronchial and vascular structures within the area affected by the pathological process. Additionally, it may be related to interstitial thickening, partial air space filling, partial alveoli collapse, increased capillary blood volume, or an association with any of these factors^(11)^. Ground-glass opacities in patients with COVID-19 pneumonia tend to be multi- and bilateral, predominantly peripheral, mainly posterior and with a slight predominance in the middle and lower pulmonary fields (Figures 1 and 2), indicated as the most frequent initial signs in most cases^(7,15-17)^. Although literature describes a variable incidence of ground-glass opacities, it is considered an usual finding, ranging between 86% and 100% in some studies^(15,18,19)^.

### Consolidation

Consolidation refers to the replacement of air in the alveolar space by cells, tissues, or pathological fluids, obscuring the local vessels and bronchial margins, sometimes forming air bronchograms^(11)^. In COVID-19, consolidations are often associated with ground-glass opacities with a similar distribution pattern^(16,17,20)^ (Figure 2). Consolidations tend to appear later, indicating the organized pathophysiological disease process, and are frequently observed after the onset of reticular opacities and crazy-paving patterns^(15,17)^. They are found to occur in 31.8% to 41% of cases^(16,17)^; however, this incidence varies, especially when evaluated together with ground-glass and/or reticular opacities^(15,18)^.

### Reticular pattern

**Figure 3:**
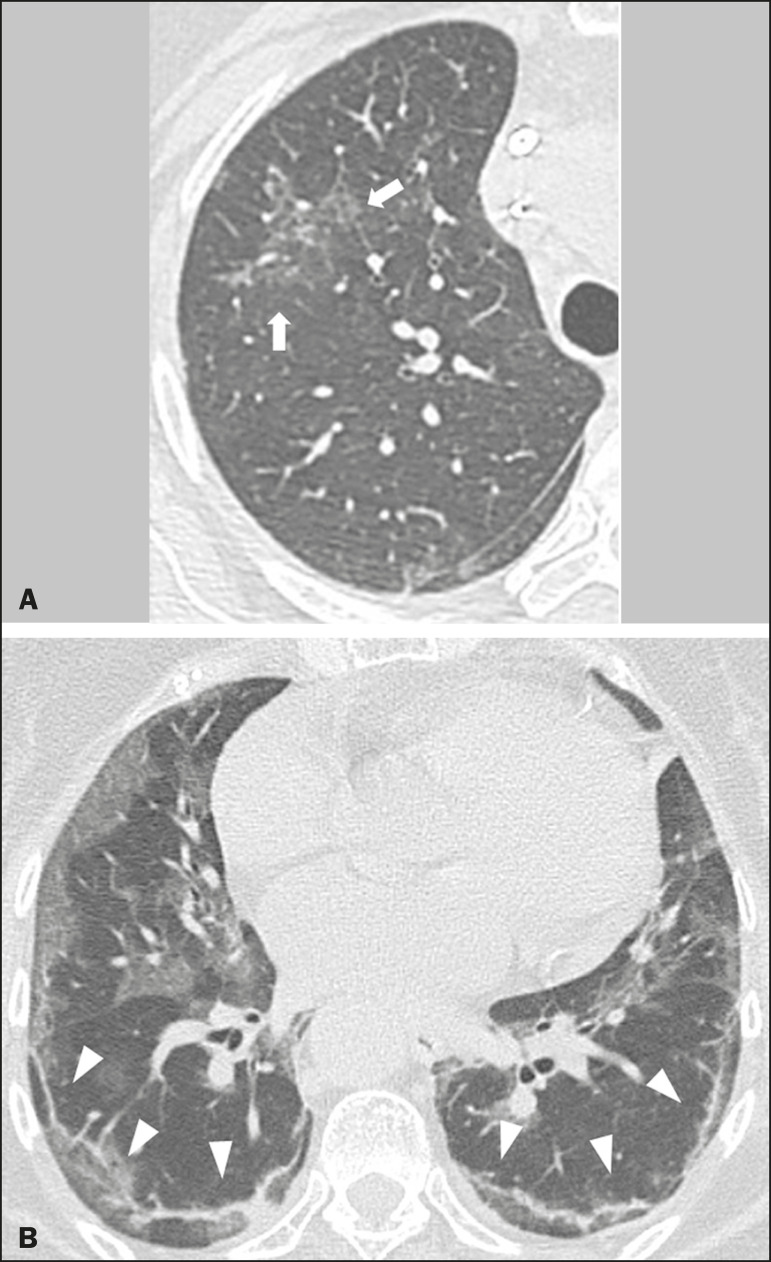
Female patients, 51 (**A**) and 68 years old (**B**). MDCT shows reticular opacities (arrows) in the anterior segment of the right upper lobe and bilateral subpleural curvilinear lines (arrowheads) in the lower lobes. Additionally, other multifocal opacities with ground-glass attenuation are present. Positive RT-PCR for SARS-CoV-2 in both patients.

Usually associated with interstitial diseases, reticular patterns demonstrate the thickening of interlobular and intralobular septa, presenting as linear opacities on tomography^(11)^. This is not a common finding in the initial presentations of COVID-19 and is observed in less than 22% cases^(15,19)^, usually in later stages of the disease and preferentially in the periphery of the lungs^(7,17,20)^ (Figure 3A). Conversely, 20% of patients with pneumonia caused by the novel coronavirus have been reported to exhibit subpleural lines, which are nonspecific indicators of atelectasis, edema, fibrosis, or inflammation, characterized by subpleural curvilinear opacities parallel to its surface^(11,18)^ (Figure 3B).

### Crazy-paving

**Figure 4:**
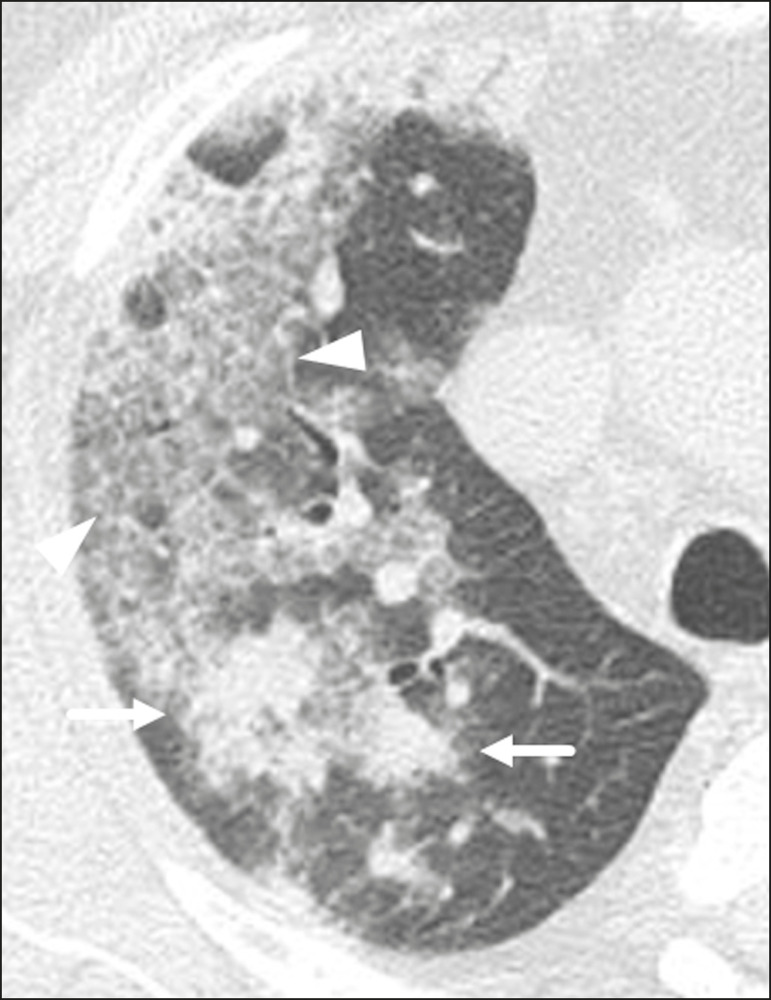
Male patient, 59 years old. MDCT shows ground-glass opacity in the right lung associated with interlobular and intralobular septal thickening, characterized by the crazy-paving pattern (arrowheads) and some consolidation foci (arrows). Positive RT-PCR for SARS-CoV-2.

Crazy-paving indicates the overlapping of ground-glass opacities and thickening of interlobular and intralobular septa, with a well-defined interface between adjacent normal lung parenchyma, indicating alveolar and interstitial involvement^(11)^. Tomographic identification suggests disease progression or even a peak stage, observed in 53% of cases^(15)^; however, it is not common during resolution phases^(15,17)^ (Figure 4).

### Reversed halo sign

**Figure 5:**
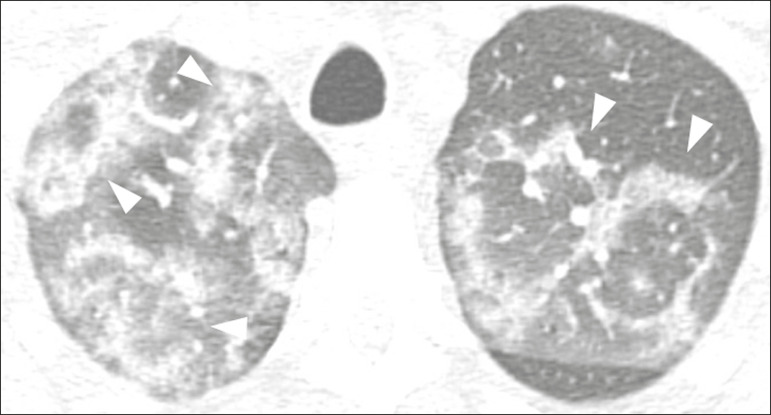
Male patient, 56 years old. MDCT shows ground-glass attenuation opacities, partially or surrounded by a consolidation ring, characterized by the reversed halo sign (arrowheads) in both lungs. Positive RT-PCR for SARS-CoV-2.

This indicates a ground-glass opacity that is partially or completely surrounded by a consolidation ring^(11)^, which may represent stages of disease progression with a pattern of organizing pneumonia^(9,17,21)^; it has been observed in up to 8% of cases^(19)^ (Figure 5).

### Nodule

Nodules are defined as rounded focal opacities measuring less than 3 cm, with or without calcifications that can be solid, partially solid, or only presenting ground-glass attenuation^(11)^. These can be observed in viral pneumonia and have been reported in 3-13% of COVID-19 pneumonia cases^(8,18,22)^, with some demonstrating a surrounding ground-glass zone that forms the halo sign^(8,23,24)^.

### Parenchymal band

**Figure 6:**
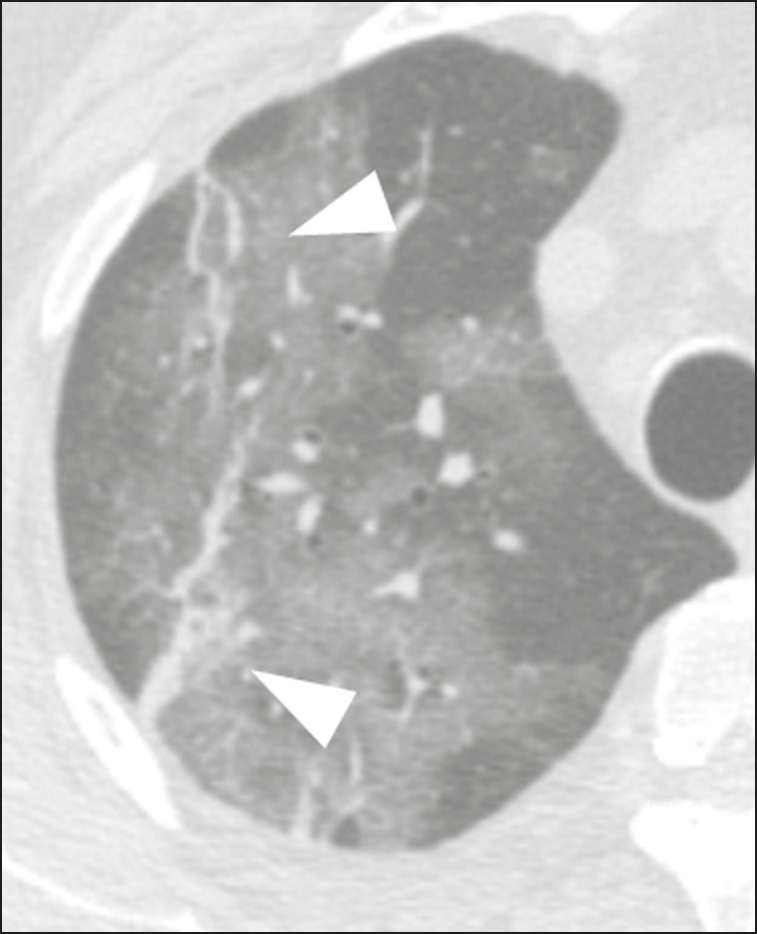
Male patient, 70 years old. MDCT shows parenchymal bands/fibroatelectatic band opacities on the pulmonary periphery. Additionally, other opacities with ground-glass attenuation are observed. Positive RT-PCR for SARS-CoV-2.

The parenchymal band is described as a peripheral linear opacity, perpendicular or oblique to the pleural surface, which may be thickened or retracted at the contact site. Furthermore, it is usually associated with pleural parenchymal fibrosis^(11)^ and occurs more frequently during advanced stages of the disease compared to initial stages^(7)^ (Figure 6). Although some studies suggest that fibrotic streaks may appear during the resolution of parenchymal aggression, with inflammatory cells replaced by fibrous tissue, other studies report that it is a sign of poor prognosis, with progression to fibrous interstitial disease^(8,15,22)^.

### Pseudo-cavity

**Figure 7:**
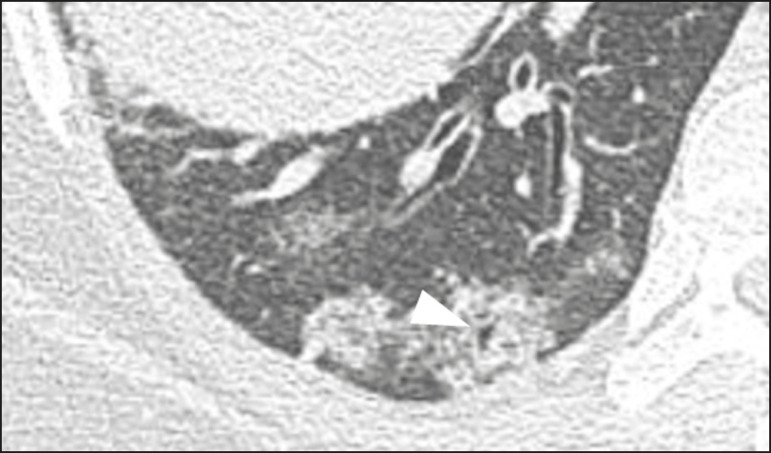
Male patient, 41 years old. MDCT shows pseudo-cavity associated with consolidation and ground-glass opacity on the right lower lobe (arrowhead). Positive RT-PCR for SARS-CoV-2.

The pseudo-cavity represents an area less than 1 cm in diameter and has a low attenuation coefficient inside pulmonary nodules/masses or consolidations^(11)^. In cases of pneumonia caused by the novel coronavirus, pseudo-cavity may represent a pathological airspace dilation, cross-sectional bronchiectasis, or a consolidation area during the resorption process^(21)^ (Figure 7). It is important to establish a differential diagnosis with cavitary lesions (cavities/excavations), which are atypical and infrequent injuries in COVID-19 pneumonia. Furthermore, associated complications such as overlapping secondary infections and alternative diagnoses should be considered^(3,16,17,25)^.

### Vascular ectasia

**Figure 8:**
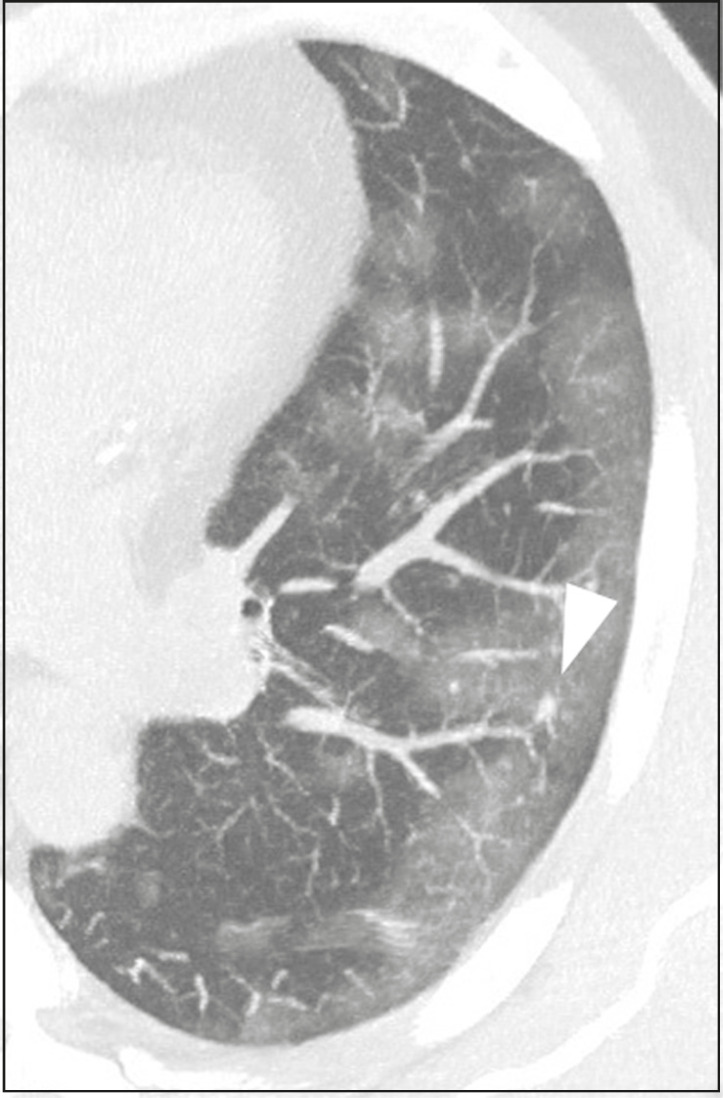
Male patient, 44 years old. Axial MDCT image with maximum intensity projection reconstruction shows extensive peripheral opacity with groundglass attenuation and some ectatic vessels inside (arrowhead). Positive RTPCR for SARS-CoV-2.

Vascular ectasia is usually represented by the dilation of pulmonary vessels inside or adjacent to a lesion due to damage and capillary edema caused by the activity of local inflammatory factors^(18)^ (Figure 8).

## PLEURAL MANIFESTATIONS

**Figure 9:**
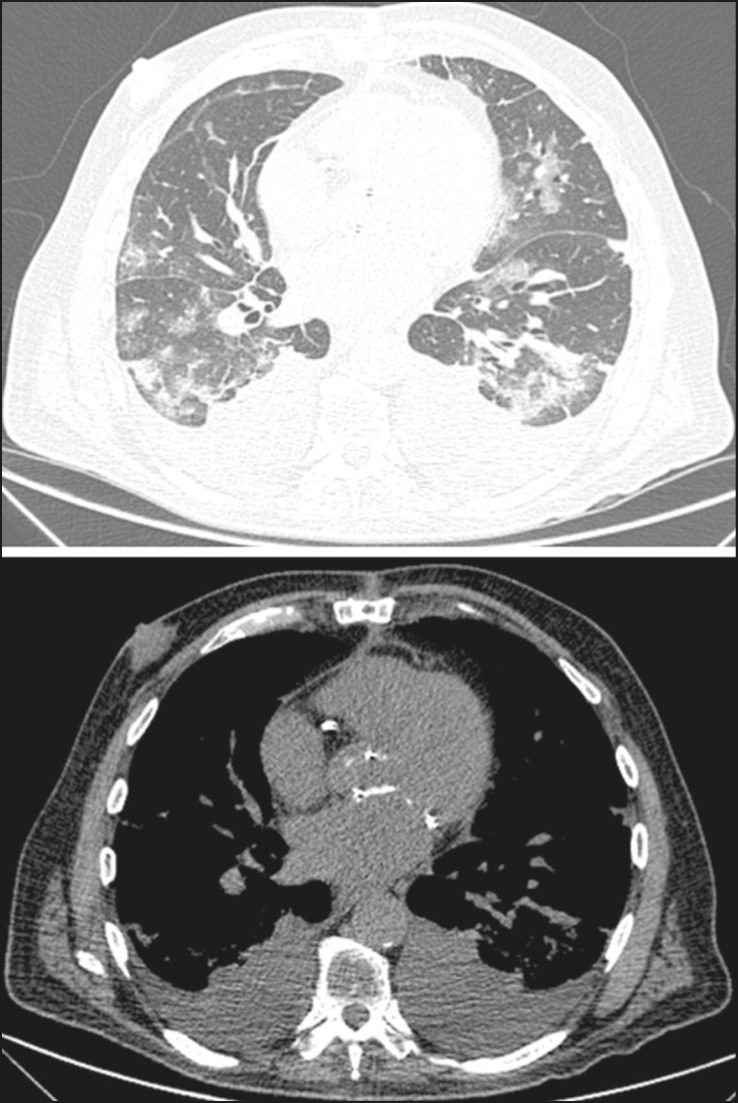
Male patient, 69 years old. MDCT shows diffuse ground-glass opacity in both lungs and consolidation foci associated with a minor bilateral pleural effusion, indicating restrictive atelectasis of the adjacent lung parenchyma. Positive RT-PCR for SARS-CoV-2.

Pleural changes are less frequent^(17,25)^, especially during the initial stages. However, pleural thickening, effusion (Figure 9), and retraction can occur during the disease^(16,20)^. The presence of pleural effusion suggests overlapping infection or alternative diagnoses^(8)^.

## AIRWAY MANIFESTATIONS

**Figure 10:**
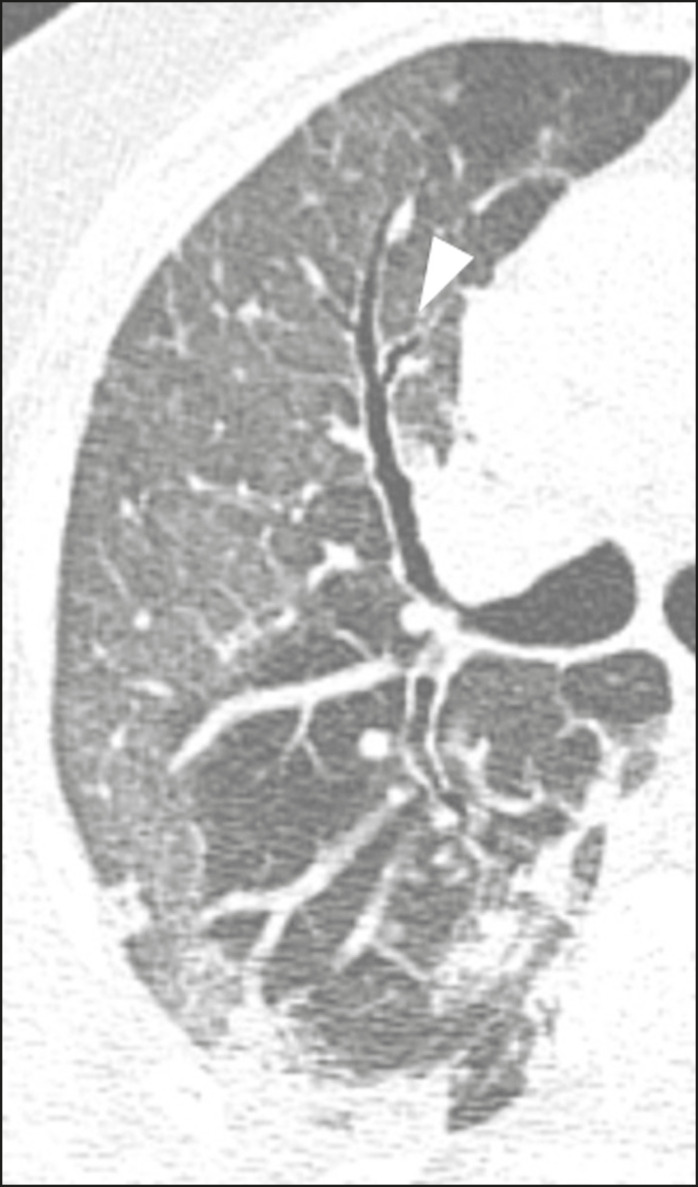
Female patient, 76 years old. MDCT shows air bronchogram between opacity with ground-glass attenuation and some consolidation foci associated with bronchial wall thickening and distortion (arrowhead). Positive RT-PCR for SARS-CoV-2.

Airway changes, including bronchial wall thickening and centrilobular micronodules and with tree-in-bud, remain infrequent. The most observed sign is an air bronchogram (Figure 10), identified between ground-glass opacities or consolidations. Peribronchial inflammatory changes, represented by parietal or peribronchovascular interstitial thickening, can retract the walls with consequent distortion and ectasia^(20)^ (Figure 10).

## CARDIOVASCULAR AND MEDIASTINAL MANIFESTATIONS

Cardiovascular complications including myocarditis, acute myocardial infarction, and exacerbation of heart failure are associated with comorbidities and other complications; such complications have been well recognized in historical viral epidemics, such as influenza and previous coronavirus outbreaks^(13,26)^, due to the mechanism of angiotensin-converting enzyme 2 and the intense cytokine production by T helper lymphocytes types 1 and 2^(13)^. The consequences of cardiac symptoms can be intensified by acute respiratory distress syndrome, as well as by mechanical ventilation with hemodynamic implications.

**Figure 11:**
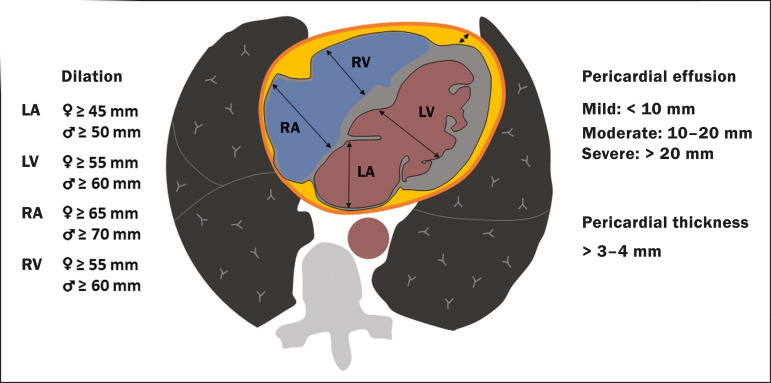
Illustrative scheme to measure the cardiac chambers and estimate the volume of pericardial effusion/thickening in chest tomography not coupled to an electrocardiogram. The left atrium (LA) must be measured in the anteroposterior diameter while the other cardiac chambers must be measured in the transverse diameter^(27,28)^.

In cases of limited availability or use of echocardiogram and/or cardiac magnetic resonance, contrasted-enhanced chest CT, even if not coupled with the electrocardiogram, can provide information regarding the diameter of cardiac chambers as well as pericardial changes. The dimensions of the cardiac chambers can be estimated using the evaluation proposed by Hota et al.^(27)^ (Figure 11). The pericardial effusion volume can be estimated using the evaluation proposed by Ivens et al.^(28)^ (Figure 11), which considers a normal pericardial thickness of up to 2 mm.

Pericardial effusion and mediastinal lymph node enlargement (diameter greater than 1 cm in its shortest axis) are uncommon findings during the initial stages of COVID- 19^(15,17,25)^; however, when present, they both suggest a more severe disease state^(29)^.

## CONCLUSION

Recent studies have suggested that chest CT has greater sensitivity but low specificity when compared to RT-PCR for the diagnosis of COVID-19^(1,8,30,31)^; however, a diagnostic confirmation with the viral test is necessary for etiological diagnosis, even with typical radiological findings^(4)^. The use of CT to evaluate patients with suspected COVID-19 has been increasingly considered, although most professional societies do not recommend its use as a screening method^(3-6)^. The predominant typical radiological findings include peripheral and bilateral multilobe and bilateral ground-glass opacities, with or without septal consolidation, round opacities with ground-glass attenuation with or without consolidation foci or septal thickening, or the reverted halo sign (or other findings of organizing pneumonia). When these imaging patterns are not present, chest CT scans can still be categorized as indeterminate, atypical, or negative for pneumonia, highlighting that some tests demonstrate no radiological changes during the earlier stages of the disease^(3)^.
